# A numerical method for computing interval distributions for an inhomogeneous Poisson point process modified by random dead times

**DOI:** 10.1007/s00422-021-00868-8

**Published:** 2021-03-19

**Authors:** Adam J. Peterson

**Affiliations:** Leibniz Institute for Neurobiology, Brenneckestrasse 6, 39118 Magdeburg, Germany

**Keywords:** Poisson point process, Inhomogeneous process, Random dead time, Interval distribution, Numerical method, Simulation

## Abstract

The inhomogeneous Poisson point process is a common model for time series of discrete, stochastic events. When an event from a point process is detected, it may trigger a random dead time in the detector, during which subsequent events will fail to be detected. It can be difficult or impossible to obtain a closed-form expression for the distribution of intervals between detections, even when the rate function (often referred to as the intensity function) and the dead-time distribution are given. Here, a method is presented to numerically compute the interval distribution expected for any arbitrary inhomogeneous Poisson point process modified by dead times drawn from any arbitrary distribution. In neuroscience, such a point process is used to model trains of neuronal spikes triggered by the detection of excitatory events while the neuron is not refractory. The assumptions of the method are that the process is observed over a finite observation window and that the detector is not in a dead state at the start of the observation window. Simulations are used to verify the method for several example point processes. The method should be useful for modeling and understanding the relationships between the rate functions and interval distributions of the event and detection processes, and how these relationships depend on the dead-time distribution.

## Introduction

The inhomogeneous Poisson point process is commonly used to model time series of discrete, stochastic events. It is applied to diverse phenomena, such as in the fields of neuroscience (e.g., Siebert 1970; Srulovicz and Goldstein 1983; Brown et al. 1998; Liu et al. 2001; Amarasingham et al. 2006), optical communications (e.g., Vannucci and Teich 1978; Drost et al. 2015; Verma and Drost 2017), and particle physics (e.g., Müller 1981b). An inhomogeneous Poisson point process has a time-varying rate and generates a sequence of events that occur at random times (Snyder 1975; Cox and Isham 1980). In the ideal case, all of the events can be observed using a suitable detector. In reality, a subset of events often fails to be observed due to a dead time in the detector (Müller 1981a; Grupen and Shwartz 2008; Picinbono 2009). One must therefore distinguish between two point processes: one of which describes the “events” themselves and the other of which describes the “detections” (i.e., the subset of events that is detected). An example from biology is the firing of spikes (i.e., action potentials) by a postsynaptic neuron in response to the release of excitatory neurotransmitter by a presynaptic neuron (with the further simplifying assumption that each neurotransmitter release event triggers a spike unless the postsynaptic neuron is in a dead state): the event process corresponds to the times at which neurotransmitter is released by the presynaptic neuron, the detection process corresponds to the times at which the released neurotransmitter triggers spikes in the postsynaptic neuron, and the dead time arises from a physiological property of neurons, known as the refractory period, that prevents spikes from being generated too soon after the previous spike (Hodgkin and Huxley 1952). An example from physics is the counting of photons by a sensor: the event process corresponds to the times at which photons arrive at the sensor, the detection process corresponds to the times at which the sensor detects photons, and the dead time arises from a property of the sensor that prevents photons from being detected if they arrive too soon after the previous detection. For simplicity, it is often assumed that all dead times in a given process have the same fixed duration (e.g., Müller 1981a; Teich 1985; Teich and Khanna 1985; Picinbono 2009), but some processes are better described by dead times with random durations drawn from a fixed distribution (e.g., Teich et al. 1978; Müller 1981a; Young and Barta 1986; Deger et al. 2010; Peterson and Heil 2018). A distinction is made between “paralyzable” and “nonparalyzable” detectors (Müller 1981a). With a paralyzable detector, an event that occurs during a dead time causes the dead time to be prolonged. With a nonparalyzable detector, an event that occurs during a dead time simply fails to be detected, but the dead time is not prolonged. Only the nonparalyzable case, as would apply to neurons, is considered here. Although a detection is synonymous with a spike in the context of a neuronal spike train, the more general term “detection” is used here to emphasize the broader applicability of the work.

**Fig. 1:**
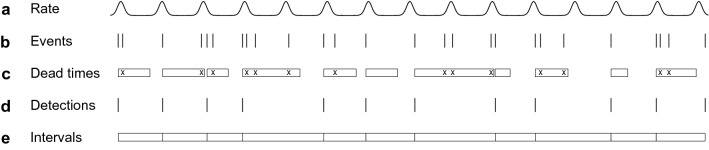
An inhomogeneous Poisson point process modified by random dead times. **a** Time-varying event rate of the point process. **b** Sequence of stochastic events generated by the process. **c** Random dead times, with each undetected event marked by an “*x*.” **d** Sequence of stochastic detections. **e** First-order intervals between detections

Figure 1 demonstrates how an inhomogeneous event process can be modified by random dead times to yield a detection process. Figure 1a shows the time-varying event rate that defines the process. This rate function (also known as the intensity function) determines the probability of an event occurring at each time point in the observation window. Figure 1b shows stochastic event times in such a process. Figure 1c shows random dead times and indicates the subset of events (marked by “x”) that fall into each dead time and are therefore not detected. For illustrative purposes, the dead times are relatively long so that many events fail to be detected. Figure 1d shows all events that do not fall into a dead time and are therefore detected. Each detection triggers the start of a new random dead time. Figure 1e shows the first-order intervals between detections. The statistical properties of point processes are often investigated by computing the distributions of such intervals (e.g., Johnson 1978; Wilbur and Rinzel 1983; Gummer 1991; Shcherbakov et al. 2005; Kroó et al. 2007; Nawrot et al. 2008; Arkani and Raisali 2015; Pommé et al. 2015). However, except in very specific cases, typically involving a homogeneous Poisson point process or other simple renewal processes, it is difficult or impossible to obtain a closed-form expression to describe the interval distribution (Picinbono 2009).

Here, a numerical method is presented for computing the interval distribution expected for any arbitrary inhomogeneous Poisson point process modified by dead times drawn from any arbitrary distribution. The interval distributions obtained with this method are intended to be analogous to distributions computed from experimental data. Because experimental data are collected over a finite observation window (or several repetitions of a finite window), empirical interval distributions are distorted by the fact that an interval between detections cannot exceed the time remaining in the observation window and therefore cannot be arbitrarily long. This distortion effect is known as right censoring and leads to an overrepresentation of short intervals and underrepresentation of long intervals (e.g., Wiener 2003; Nawrot et al. 2008). Nonetheless, to maintain equivalence with experimentally obtained interval distributions, the numerical method must also reproduce the right censoring caused by the finite observation window. The numerical method presented here does this and is therefore applicable to a variety of experimentally observed phenomena in several fields of study.

## Methods

The numerical method presented below was implemented in MATLAB R2019b and is publicly available in an online code repository (Peterson 2020). The method has been divided into several algorithms, with the goal of presenting each algorithm as straightforwardly as possible without regard for computational demand. Those algorithms found to be computationally demanding were then reimplemented using more optimized but less comprehensible MATLAB code. One particularly demanding computation (Eq. 8) was reimplemented using C code and compiled to a MATLAB MEX file to enable faster execution. All implementations of each algorithm are included in the online repository, along with a demonstration of their equivalence.

The following notations and conventions are used throughout:

1. The observation window of the point process spans the interval from *t*_min_ to *t*_max_ and is divided into bins of width Δ*t.* Each bin is referenced by the time point at its right edge, denoted *t*_*i*_, where *i* is the index of the bin within the observation window. For an observation window starting at *t*_min_ = 0 s, the time point marking the first bin is *t*_1_ = Δ*t*.
2. Durations in the dead-time distribution are denoted *d*_*j*_, where *j* is the index of a bin within the distribution. For the numerical method presented here, the shortest duration that needs to be considered is *d*_1_ = Δ*t*.
3. Waiting times in the forward recurrence distributions or intervals in the interval distributions are denoted *w*_*k*_, where *k* is the index of a bin within the distribution. For the numerical method presented here, the shortest waiting time or interval that needs to be considered is *w*_1_ = Δ*t*.
4. For simplicity, the bin indices *i*, *j*, and *k* are omitted from the figures, captions, and occasionally elsewhere.

Before describing each step of the numerical method below, it is necessary to define several prerequisite functions. For clarity, all prerequisites and algorithm steps are demonstrated using one example point process of events and one example dead-time distribution.

### Prerequisite 1: The rate (or probability) function of the detection process

**Fig. 2:**
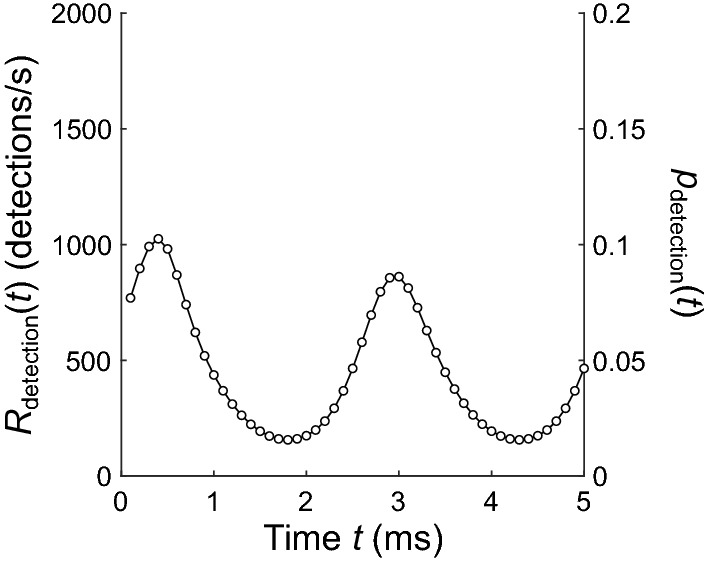
Example inhomogeneous detection rate over an observation window with *t*_min_ = 0 ms, *t*_max_ = 5 ms, and Δ*t* = 0.1 ms. The rate can be scaled to yield detection probability *p*_detection_ (right axis)

To compute the interval distribution expected for an inhomogeneous Poisson point process modified by random dead times, it is necessary to know the rate of the detection process over the observation window (i.e., the rate obtained from experimental measurements). The numerical method presented here will work for any arbitrary rate function. Figure 2 shows the example rate *R*_detection_ (in detections/s) of the detection process used to demonstrate the method. Here, the observation window is 5 ms long and the time step is Δ*t* = 0.1 ms. Note that multiplying the rate function by Δ*t* (in seconds) will convert it to an equivalent probability function *p*_detection_, which is also shown (right axis).

### Prerequisite 2: The distribution of dead times

To compute the interval distribution expected for an inhomogeneous Poisson point process modified by random dead times, it is also necessary to know the distribution from which the dead times are drawn. The numerical method presented here will work for any arbitrary distribution, as long as the random dead times are independent and drawn from the same distribution. Throughout the demonstrations below, the dead time is a random variable *d*_dead_ equal to the sum of a fixed duration *d*_fixed_ and a random duration *d*_rand_ drawn from the geometric distribution with a mean of *μ*_rand_. This is the discrete equivalent of a continuous distribution of dead times consisting of a fixed portion and a random portion drawn from the exponential distribution, such as was used by Young and Barta (1986) and Peterson and Heil (2018). The cumulative distribution function (CDF) for *d*_dead_ (i.e., the probability that *d*_dead_ is shorter than or equal to duration *d*_*j*_) is given by

1 $$  G_{\text{dead}} \left( d_{j} \right)= \left\{\begin{array}{*{20}l}1 - \left( {1 - \frac{{{\Delta }t}}{{\mu_{{{\text{rand}}}} }}} \right)^{{\left( {d_{j} - d_{{{\text{fixed}}}} } \right)/{\Delta }t}} \hfill & {\text{for}}\;d_{j} \ge d_{{{\text{fixed}}}} \hfill \\  0 \hfill & {\text{for}}\;d_{j} < d_{{{\text{fixed}}}} \hfill \\ \end{array}\right.$$

**Fig. 3:**
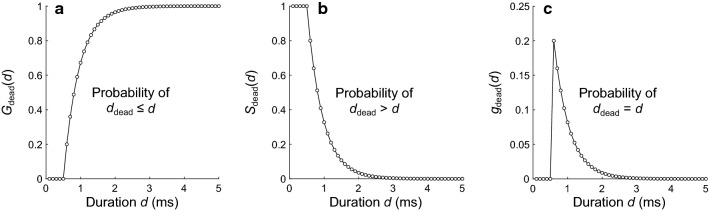
Example dead-time distribution, where *d*_dead_ is the random variable of dead times. **a** The CDF for *d*_dead_, given by Eq. 1. **b** The survivor function for *d*_dead_, given by Eq. 2. **c** The PMF for *d*_dead_, given by Eq. 3. For all three functions, *d*_fixed_ = 0.5 ms, *μ*_rand_ = 0.5 ms, and Δ*t* = 0.1 ms

This distribution is equivalent to the cumulative geometric distribution $G(k)=1-{\left(1-p\right)}^{k}$ (with success probability *p* and trial *k* ∈ {1, 2, 3,…}) but is parameterized in terms of time and is delayed by *d*_fixed_. Figure 3a shows the CDF for the example dead-time distribution (with *d*_fixed_ = 0.5 ms and *μ*_rand_ = 0.5 ms). The survivor function for *d*_dead_ (i.e., the probability that *d*_dead_ is longer than duration *d*_*j*_) is given by the complement of the CDF,

2 $$ S_{\text{dead}} \left( d_{j}  \right)  = \left\{ {\begin{array}{*{20}l}1 - G_{{{\text{dead}}}} \left( {d_{j} } \right) = \left( {1 - \frac{{{\Delta }t}}{{\mu_{{{\text{rand}}}} }}} \right)^{{\left( {d_{j} - d_{{{\text{fixed}}}} } \right)/{\Delta }t{ }}} \hfill & {\text{for}}\;d_{j} \ge d_{{{\text{fixed}}}} \hfill \\  1\hfill & {\text{for}}\;d_{j} < d_{{{\text{fixed}}}} \hfill \\ \end{array} } \right.$$

Figure 3b shows the survivor function for the example dead-time distribution.

The probability mass function (PMF) for *d*_dead_ (i.e., the probability that *d*_dead_ is equal to duration *d*_*j*_) is given by

3 $$ g_{\text{dead}} \left(d_{j} \right) =\left\{ {\begin{array}{*{20}l} \left( {\frac{{{\Delta }t}}{{\mu_{{{\text{rand}}}} }}} \right) \cdot \left( {1 - \frac{{{\Delta }t}}{{\mu_{{{\text{rand}}}} }}} \right)^{{\left( {d_{j} - d_{{{\text{fixed}}}} } \right)/{\Delta }t{ } - 1}} \hfill & {\text{for}}\;d_{j} > d_{{{\text{fixed}}}} \hfill \\  0 \hfill & {\text{for}}\;d_{j} \le d_{{{\text{fixed}}}}\hfill  \\ \end{array} } \right.$$

Figure 3c shows the PMF for the example dead-time distribution. Note that, as the time step decreases toward 0 ms, the PMF approaches the continuous probability density function (PDF) for a dead time having a random portion drawn from the exponential distribution (not shown).

With the rate of the detection process and the dead-time distribution specified, it is now possible to proceed to the first step in the numerical method.

### Step 1: Computing the rate (or probability) function of the event process

**Fig. 4:**
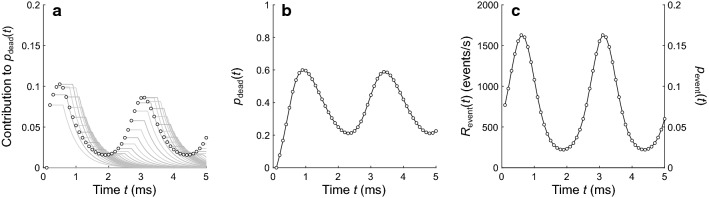
Computing the event rate. **a** Each time point (marked by symbols) contributes to the probability *p*_dead_ of the detector being in a dead state during all time points that follow. The contribution of each time point (gray lines) is given by the dead-time survivor function *S*_dead_ (Fig. 3b) scaled by probability *p*_detection_ of a detection at the preceding time point. **b** The overall *p*_dead_ function during the observation window, computed with Eq. 4, is equivalent to the binwise sum of the individual contributions in **a**. **c** The event probability *p*_event_ (right axis) computed with Eq. 6, which can be scaled to yield the event rate *R*_event_ (left axis)

For the numerical method presented here, it is necessary to know the probability *p*_event_ of an event at each time point in the observation window. If the dead-time distribution is known, then it is possible to compute *p*_event_ from *p*_detection_. This computation requires knowing the probability *p*_dead_ that the detector is in a dead state at each time point in the observation window, given by

4 $$  p_{\text{dead}} \left( t_{i}  \right) =\left\{ {\begin{array}{*{20}l} 0 \hfill & {\text{for}}\;i = 1 \hfill \\ \mathop \sum \limits_{h = 1}^{i - 1} p_{{{\text{detection}}}} \left( {t_{h} } \right) \cdot S_{{{\text{dead}}}} \left( {d_{i - h} } \right) \hfill & {\text{for}}\;i \ge 2 \hfill \\ \end{array} } \right. $$

where *S*_dead_(*d*) is the survivor function for the dead times (i.e., the probability that *d*_dead_ is longer than duration *d*), *t*_*h*_ = *h*⋅Δ*t* is a time point of a bin preceding bin *i*, and *d*_*i-h*_ = (*i*−*h)*⋅Δ*t* is a dead-time duration. For simplicity, the process is assumed to be free from dead-time effects at the first time point in the observation window. For each subsequent time point *t*_*i*_, the value of *p*_dead_(*t*_*i*_) can be obtained from the probability *p*_detection_ of a detection at each previous time point *t*_*h*_ and the probability *S*_dead_(*d*_*i-h*_) that the dead time following each previous detection would extend through time point *t*_*i*_. More precisely, the contribution of a previous time point *t*_*h*_ to *p*_dead_ at each subsequent time point *t*_*i*_ is given by the joint probability *p*_detection_(*t*_*h*_)·*S*_dead_(*d*_*i-h*_). Figure 4a shows how each time point in the example process (marked by a symbol) contributes to *p*_dead_ at all later time points (corresponding gray line). The summation of the joint probabilities in Eq. 4 is equivalent to the binwise sum of the individual joint probability functions in Fig. 4a*.* Figure 4b shows the resultant *p*_dead_(*t*_*i*_) computed for the example process. Note that Eq. 4 is nothing other than the convolution of *p*_detection_ and *S*_dead_ and could be replaced by a more efficient algorithm such as convolution based on the fast Fourier transform.

Recall that two conditions must be met for a detection to occur: an event must occur, and the detector must not be in a dead state. The detection probability *p*_detection_(*t*_*i*_) is therefore given by the joint probability

5 $$ p_{{{\text{detection}}}} \left( {t_{i} } \right) = p_{{{\text{event}}}} \left( {t_{i} } \right) \cdot \left( {1 - p_{{{\text{dead}}}} \left( {t_{i} } \right)} \right) $$

Equation 5 can be rearranged to obtain the probability *p*_event_ of an event at each time point in the observation window,

6 $$ p_{{{\text{event}}}} \left( {t_{i} } \right) = \frac{{p_{{{\text{detection}}}} \left( {t_{i} } \right)}}{{\left( {1 - p_{{{\text{dead}}}} \left( {t_{i} } \right)} \right)}} $$

Figure 4c shows *p*_event_(*t*_*i*_) computed for the example process. It is higher than *p*_detection_(*t*_*i*_) (Fig. 2) at all time points except the first one. At the first point, *p*_event_ and *p*_detection_ are identical because it is assumed that the detector is not in a dead state at the start of the observation window (i.e., it is assumed that *p*_dead_(*t*_1_) = 0). Note that Eq. 6 is undefined in the unlikely case that *p*_dead_(*t*_*i*_) = 1, which would occur only when *t*_*i*_ falls into the fixed portion of a dead time following a time point for which a detection is guaranteed to occur (i.e., for which *p*_detection_ = 1). In such a case, *p*_detection_(*t*_*i*_) = 0 regardless of the value of *p*_event_(*t*_*i*_), and the event probability would therefore be unknowable and unrecoverable.

Note that if *p*_event_ were known, rather than *p*_detection_, then *p*_dead_ and *p*_detection_ could be computed using Eqs. 4 and 5. For all time points *t*_*i*_ following the initial point, the computation of *p*_dead_ (Eq. 4) depends on the value of *p*_detection_ from all previous time points *t*_*h*_, whereas the computation of *p*_detection_ (Eq. 5) depends on *p*_dead_ from the current time point *t*_*i*_. This interdependence of *p*_dead_ and *p*_detection_ requires that both values be computed at a given time point before proceeding to the next point. For the numerical method presented here, it is necessary to know both *p*_event_ and *p*_detection_, no matter which of the two was known initially.

### Step 2: Computing forward recurrence distributions for events

The numerical method presented here makes heavy use of forward recurrence distributions. The distribution of forward recurrence times, *f*_event_(*t*_*i*_,*w*_*k*_), specifies the probability that, at time point *t*_*i*_, the waiting time (or recurrence interval) to the next event is equal to *w*_*k*_. More precisely, the probability that the next event will occur with a waiting time of *w*_*k*_ equals the joint probability that there is an event at time *t*_*i*_ + *w*_*k*_ and that there are no events between times *t*_*i*_ and *t*_*i*_ + *w*_*k*_. At time point *t*_*i*_ in the observation window, the forward recurrence probability *f*_event_ that the waiting time to the next event is equal to *w*_*k*_ is given by

7 $$ f_{{{\text{event}}}} \left( {t_{i} ,w_{k} } \right) = \left\{ {\begin{array}{*{20}l} { p_{{{\text{event}}}} \left( {t_{i} + w_{k} } \right)} \hfill & {{\text{for }}k = 1} \hfill \\ { p_{{{\text{event}}}} \left( {t_{i} + w_{k} } \right) \cdot  \mathop \prod \limits_{h = i + 1}^{i + k - 1} \left( {1 - p_{{{\text{event}}}} \left( {t_{h} } \right)} \right)} \hfill & {{\text{for }}k \ge 2} \hfill \\ \end{array} } \right. $$

**Fig. 5:**
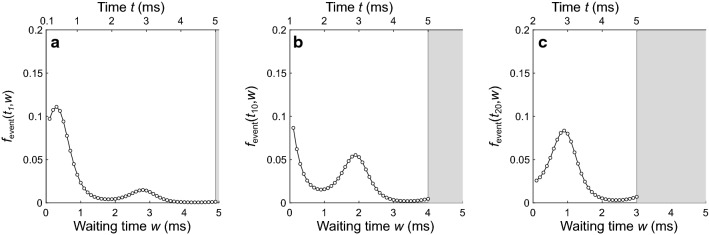
Example forward recurrence distributions, *f*_event_(*t*,*w*), showing the probability that, at time point *t* in the process, the waiting time to the next event will equal *w*. **a** *f*_event_ for time *t*_1_ = 0.1 ms. **b** *f*_event_ for time *t*_10_ = 1 ms. **c** *f*_event_ for time *t*_20_ = 2 ms. Each distribution is computed with Eq. 7. The top axis in each panel shows the time relative to the 5-ms observation window. Gray shading marks the region missing from each distribution (i.e., the region that would extend beyond the edge of the observation window). Each distribution is therefore incomplete

Here, for each time point *t*_*i*_, the product given by the ∏ notation computes the survivor function of the event process over the waiting times *w*_*k*_. Figure 5 shows selected forward recurrence distributions computed for the example process in Fig. 2. Figure 5a shows the forward recurrence distribution *f*_event_ computed for the first time point (*t*_1_ = 0.1 ms), Fig. 5b shows *f*_event_ computed for the tenth time point (*t*_10_ = 1 ms), and Fig. 5c shows *f*_event_ computed for the twentieth time point (*t*_20_ = 2 ms). For each recurrence distribution, potential waiting times range from as short as a single bin (*w*_1_ = Δ*t*) to as long as the total number of bins remaining in the observation window (*w*_max_ = *t*_max_ − *t*_*i*_). Recurrence probabilities are not needed for waiting times that would extend the process beyond the edge of the observation window and are omitted; each distribution shown is therefore incomplete because the sum of its probabilities will be less than 1. For each recurrence distribution in Fig. 5, the upper time axis shows the corresponding time within the observation window. The forward recurrence distribution in Fig. 5a is one bin shorter than the observation window, with this edge marked by the gray shading beginning at *w* = 4.9 ms (i.e., *t* = 5 ms on the upper axis). As *t*_*i*_ increases, the corresponding forward recurrence distribution becomes increasingly incomplete. Although results are shown for only three example time points, the method requires *f*_event_ to be computed for all time points in the observation window except for the final point (there is no recurrence possible after the final point, so there is no need to compute its recurrence distribution here). Note that when the event rate is periodic, as in the example (Fig. 4c), the distributions of forward recurrence times to the next event are identical for any two points separated by an integer number of periods, except that the distribution for the later point is more incomplete. This means that, for periodic event rates, the forward recurrence distributions only need to be computed for the points within the first cycle in the observation window; they can then be duplicated (incompletely) to obtain corresponding distributions for the points in each subsequent cycle. For aperiodic event rates, the forward recurrence distributions must be computed for all points in the observation window.

### Step 3: Computing forward recurrence distributions for detections

Due to the random dead times in the detection process, the distribution of forward recurrence times to the next detection cannot be computed as straightforwardly as the distribution of forward recurrence times to the next event. In the method presented here, recurrence times to the next detection after time *t*_*i*_ are computed under the assumption that a detection has occurred at time *t*_*i*_. Note that, with the assumption of a detection at *t*_*i*_, the forward recurrence times actually represent intervals (unlike forward recurrence times in the event process, for which there was no assumption of an event at time *t*_*i*_). Assuming a detection at time *t*_*i*_, the distribution of forward recurrence times to the next detection can be computed by considering the effect of each possible dead-time duration. If the dead time following a detection at *t*_*i*_ ends after duration *d*_*j*_ (i.e., at time *t*_*i*+*j*_ = *t*_*i*_ + *d*_*j*_), two facts are apparent about the distribution of forward recurrence times to the next detection, *f*_detection_(*t*_*i*_,*w*_*k*_). First, for any waiting time *w*_*k*_ shorter than *d*_*j*_, the recurrence probability *f*_detection_ = 0. Second, for any waiting time *w*_*k*_ longer than or equal to *d*_*j*_, the recurrence probability *f*_detection_ must follow the time course of *f*_event_ (Eq. 7) computed for time point *t*_*i*+*j*_ (i.e., for the first point in the observation window free from the effects of the dead time). More specifically, the joint probability that a detection at time *t*_*i*_ is followed by a dead time with duration *d*_*j*_ (i.e., ending at time *t*_*i*+*j*_) and that the next detection occurs after waiting time *w*_*k*_ following the detection is given by *g*_dead_(*d*_*j*_)·*f*_event_(*t*_*i*+*j*_,*w*_*k*_). For each time point in the observation window, such a joint probability function is computed over all remaining bins. For time point *t*_*i*_, the forward recurrence probability *f*_detection_ is obtained by summing the respective contributions from each of the joint probability functions,

8 $${f}_{\mathrm{detection}}\left(t_i,w_k\right)=\sum_{j=1}^{k}{g}_{\mathrm{dead}}\left(d_j\right)\cdot f_{\text{event}}\left(t_{i+j-1},w_{k-j+1}\right)$$

**Fig. 6:**
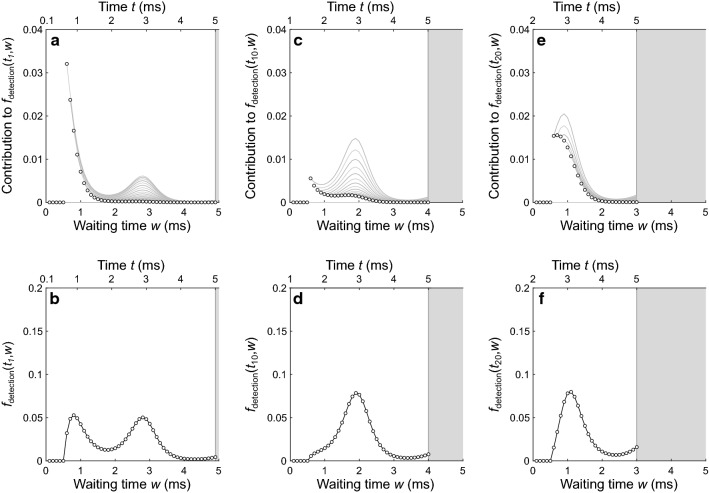
Example forward recurrence distributions, *f*_detection_(*t*,*w*), showing the probability that, given a detection at time point *t* in the process, the waiting time to the next detection will equal *w*. **a** Contributions to *f*_detection_ following a detection at time *t*_1_ = 0.1 ms. Each possible waiting time (marked by symbols) contributes to *f*_detection_ for all time points that follow. The contribution of each waiting time *w* (gray lines) is given by the portion of *f*_event_ (Fig. 5a) over the interval [*w, w*_max_], scaled by the probability *g*_dead_ that the dead time has duration *d* = *w* (Fig. 3c). **b** The overall *f*_detection_ function for *t*_1_ = 0.1 ms, computed with Eq. 8, is equivalent to the binwise sum of all individual contributions in **a**. **c** Contributions to *f*_detection_ following a detection at time *t*_10_ = 1 ms. **d** The overall *f*_detection_ function for *t*_10_ = 1 ms. **e** Contributions to *f*_detection_ following a detection at time *t*_20_ = 2 ms. **f** The overall *f*_detection_ function for *t*_20_ = 2 ms. The top axis in each panel shows the time relative to the 5-ms observation window. Gray shading marks the region missing from each distribution (i.e., the region that would extend beyond the edge of the observation window). Each distribution is therefore incomplete

Here, *w*_*k*_ is the waiting time following a detection at time *t*_*i*_ and *g*_dead_(*d*_*j*_) is the probability that the dead time *d*_dead_ has duration *d*_*j*_ (Eq. 3 and Fig. 3c). Figure 6 shows selected results for the example process. Figure 6a shows all joint probability functions contributing to the forward recurrence distribution for the first time point (*t*_1_ = 0.1 ms), each of which originates at a time point marked by a symbol. Figure 6b shows the corresponding *f*_detection_, equivalent to the binwise sum of all functions in Fig. 6a (note that the vertical axes differ in scaling). Figure 6c, d shows the contributing joint probability functions and *f*_detection_ for the tenth time point (*t*_10_ = 1 ms), and Fig. 6e, f shows the contributing joint probability functions and *f*_detection_ for the twentieth time point (*t*_20_ = 2 ms). Although results are shown for only three example time points, the method requires *f*_detection_ to be computed for all time points in the observation window except for the final point (there is no recurrence possible after the final point, so there is no need to compute its recurrence distribution here). Note that when the event rate is periodic, as in the example (Fig. 4c), the forward recurrence distributions only need to be computed for the points within the first cycle in the observation window and can then be duplicated (incompletely) to obtain corresponding distributions for the points in each subsequent cycle.

### Step 4: Computing the interval distributions for the event process and for the detection process

It is now possible to compute the distribution of interevent intervals (IEIs) and the distribution of interdetection intervals (IDIs). The former requires knowing *p*_event_(*t*_*i*_) (Eq. 6 and Fig. 4c) and the distribution of forward recurrence times to the next event for each time point in the observation window*, f*_event_(*t*_*i*_,*w*_*k*_) (Eq. 7 and Fig. 5). The latter requires knowing *p*_detection_(*t*_*i*_) (Eq. 5 and Fig. 2) and the distribution of forward recurrence times to the next detection for each time point in the observation window*, f*_detection_(*t*_*i*_,*w*_*k*_) (Eq. 8 and Fig. 6). For an observation window having *m* time points, the probability *p*_IEI_ of observing an IEI with duration *w*_*k*_ is given by

9 $$p_{\text{IEI}}\left(w_k\right)=\frac{\sum_{i=1}^{m-1}{p}_{\text{event}}\left(t_i\right)\cdot {f}_{\text{event}}\left(t_i,w_k\right)}{{n}_{\text{IEIs}}}$$

Here, *n*_IEIs_ = *n*_events_ − 1 + *p*_zero_ is the expected number of IEIs per observation window, with *n*_events_ being the expected number of events per observation window and *p*_zero_ being the probability that an observation window contains zero events. Note that if every observation window were guaranteed to contain at least one event, then the expected number of IEIs would simply be *n*_IEIs_ = *n*_events_ − 1. However, observation windows containing zero events typically can occur and will yield zero IEIs rather than zero minus one IEIs, such that some portion of the 1 which was subtracted must be added back. This portion is equal to the probability that an observation window contains zero events. Here, ${n}_{\text{events}}=\left({t}_{\text{max}}-{t}_{\text{min}}\right)\cdot\left(\bar{p}_{\text{event}}/\Delta t\right)$ and ${p}_{\text{zero}}=\prod_{i=1}^{m}\left(1-{p}_{\text{event}}\left(t_i\right)\right)$, with *t*_max_ − *t*_min_ being the duration of the observation window (in seconds), $\bar{p}_{\mathrm{event}}$ being the mean event probability in the observation window, and Δ*t* being the time step (in seconds). Note that if *p*_event_ is a row vector with column index *i* and *f*_event_ is a matrix with row index *i* and column index *k*, then *p*_IEI_ in Eq. 9 is given by the matrix product of *p*_event_ and *f*_event_, normalized by *n*_IEIs_.

The corresponding probability *p*_IDI_ of observing an IDI with duration *w*_*k*_ is given by

10 $${p}_{\mathrm{IDI}}\left(w_k\right)=\frac{\sum_{i=1}^{m-1}{p}_{\mathrm{detection}}\left(t_i\right)\cdot{f}_{\mathrm{detection}}\left(t_i,w_k\right)}{{n}_{\mathrm{IDIs}}}$$

Here, *n*_IDIs_ = *n*_detections_ − 1 + *p*_zero_ is the expected number of IDIs per observation window, with ${n}_{\mathrm{detections}}=\left({t}_{\mathrm{max}}-{t}_{\mathrm{min}}\right)\cdot \left(\bar{p}_{\mathrm{detection}}/\Delta t\right)$. The assumption that the detector is not in a dead state at the start of the observation window means the probability that an observation window contains zero detections is identical to the probability that it contains zero events: ${p}_{\mathrm{zero}}=\prod_{i=1}^{m}\left(1-{p}_{\mathrm{event}}\left(t_i\right)\right)$. Note that if *p*_detection_ is a row vector with column index *i* and *f*_detection_ is a matrix with row index *i* and column index *k*, then *p*_IDI_ in Eq. 10 is given by the matrix product of *p*_detection_ and *f*_detection_, normalized by *n*_IDIs_.

The IEI distribution (in the form of a probability mass function as in Eq. 9) can be converted to rate *R*_IEI_ (in IEIs/s), by dividing each probability by the time step Δ*t* (in seconds):

11 $${R}_{\mathrm{IEI}}\left(w_k\right)=\frac{{p}_{\mathrm{IEI}}\left(w_k\right)}{\Delta t}$$

Naturally, the IDI distribution (Eq. 10) can be scaled in the same way to yield rate *R*_IDI_ (in IDIs/s):

12 $${R}_{\mathrm{IDI}}\left(w_k\right)=\frac{{p}_{\mathrm{IDI}}\left(w_k\right)}{\Delta t}$$

Numerically computed IEI and IDI distributions are shown below for the example process and several additional processes. Note that, in the context of a neuronal spike train, the IDI distribution can be equated with the interspike interval (ISI) distribution.

## Results

The correctness of the method presented above will now be demonstrated for several point processes by comparing results obtained with the numerical method to results computed from stochastic simulations. Although relatively high rates are used in these demonstrations, the numerical method yields equally precise results for low rates.

### Inhomogeneous periodic Poisson point process modified by random dead times

**Fig. 7:**
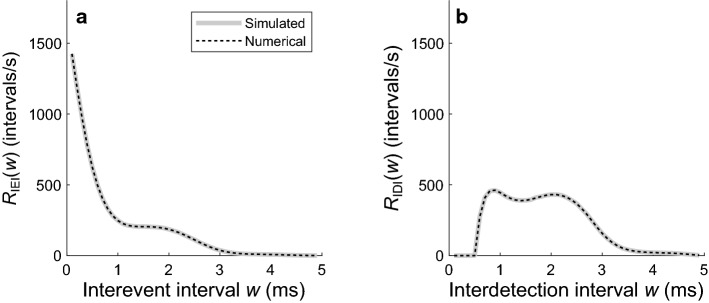
Comparison of numerical and simulation results for the example inhomogeneous Poisson point process with a periodic event rate given by Eq. 13 with *A* = 600 events/s, *B* = 1, and *f* = 400 Hz. **a** IEI distributions computed from one million simulations (gray line) and from the numerical method (dashed black line). **b** IDI distributions computed from simulations and from the numerical method after taking the dead-time effects into consideration, with *d*_fixed_ = 0.5 ms and *μ*_rand_ = 0.5 ms

For the example inhomogeneous Poisson point process above (see Methods and Figs. 2, 3, 4, 5, 6), the event rate *R*_event_ (in events/s) is given by a sinusoid passed through an exponential function,

13 $${R}_{\mathrm{event}}\left({t}_{i}\right)=A\cdot {e}^{B\cdot \mathrm{sin}\left(2\pi \cdot f\cdot {t}_{i}\right)}$$

where *A* is a scale factor (in events/s) that specifies the event rate when the instantaneous value of the sinusoid equals zero, *B* is a slope factor, *f* is the frequency (in Hertz) of the sinusoid, and *t*_*i*_ is a time point (in seconds) in the observation window. For the example process in Fig. 4c, A = 600 events/s, *B* = 1, and *f* = 400 Hz. Figure 7a shows an IEI distribution computed from one million simulations of the event process (gray line) and the IEI distribution obtained using the numerical method (dashed black line). Figure 7b shows the corresponding IDI distributions of the detection process. For each interval distribution, the numerical result and the simulation result are in close agreement. The other functions obtained using the numerical method (i.e., *p*_detection_*, p*_dead_, *f*_event_, and *f*_detection_) are in correspondingly close agreement with the simulation results (not shown). These results show that the numerical method works correctly for the example process having a periodic event rate.

### Inhomogeneous random-walk Poisson point process modified by random dead times

**Fig. 8:**
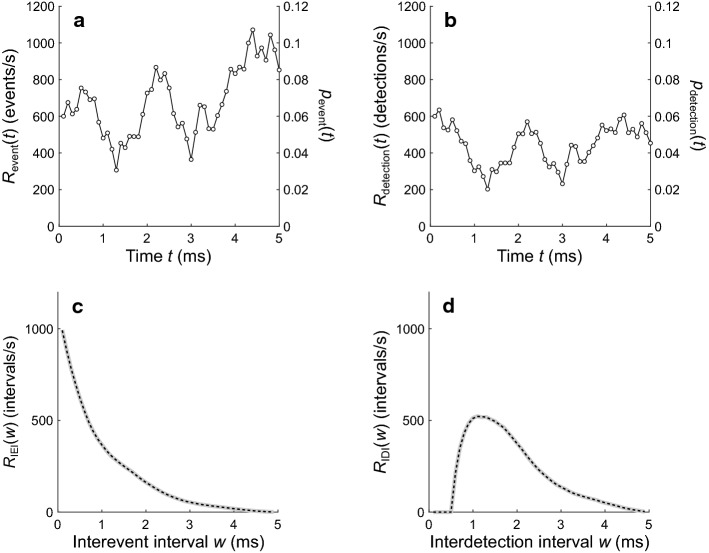
Comparison of numerical and simulation results for an inhomogeneous Poisson point process with an aperiodic event rate equal to one realization of a random walk. **a** The particular random walk used for this example. **b** The expected detection rate after taking the dead-time effects into consideration, with *d*_fixed_ = 0.5 ms and *μ*_rand_ = 0.5 ms. **c** IEI distributions computed from one million simulations (gray line) of the same random walk shown in **a**, and from the numerical method (dashed black line). **d** IDI distributions computed from the simulations and from the numerical method

Figure 8 shows results for an inhomogeneous Poisson point process whose event rate over the observation window is aperiodic and equal to one realization of a random walk. Figure 8a shows the particular random walk used for this example. The initial rate at time *t*_1_ was 600 events/s, and the rates at later time points were obtained by cumulatively summing random values from the uniform distribution on [− 75, 75] events/s. Figure 8b shows the detection rate expected if this process were modified by dead times drawn from the example distribution (Fig. 3c). Figure 8c shows an IEI distribution computed from one million simulations of the event process (gray line) and the IEI distribution obtained using the numerical method (dashed black line). Figure 8d shows the corresponding IDI distributions. The numerical results and the simulation results are in close agreement.

### Homogeneous Poisson point process modified by random dead times

**Fig. 9:**
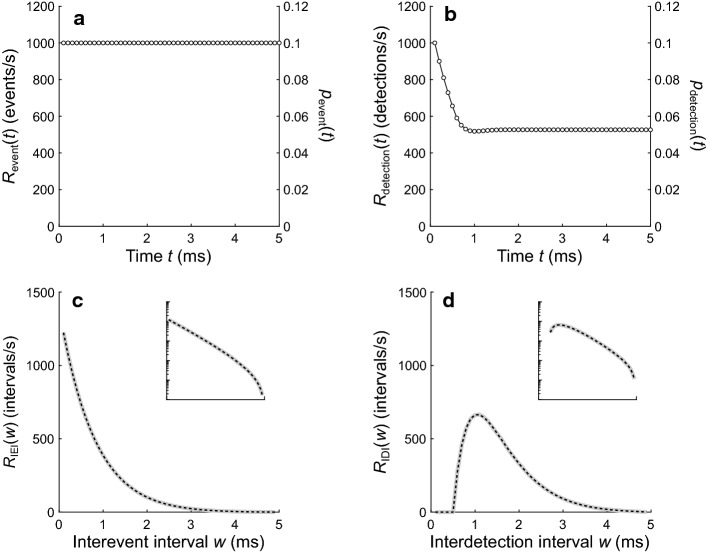
Comparison of numerical and simulation results for a homogeneous event process. **a** The constant event rate of *R*_event_ = 1000 events/s. **b** The expected detection rate after taking the dead-time effects into consideration, with *d*_fixed_ = 0.5 ms and *μ*_rand_ = 0.5 ms. **c** IEI distributions computed from one million simulations (gray line) and from the numerical method (dashed black line). **d** IDI distributions computed from the simulations and from the numerical method. Insets in **c** and **d** show the same distributions with a logarithmic rate axis

Figure 9 shows results for a homogeneous Poisson point process. Figure 9a shows its constant event rate of 1000 events/s. Figure 9b shows the detection rate expected if the process were modified by dead times drawn from the example distribution (Fig. 3c). Figure 9c shows an IEI distribution computed from one million simulations of the event process (gray line) and the IEI distribution obtained using the numerical method (dashed black line). The IEIs in the homogeneous Poisson point process have a geometric distribution (or, equivalently, an exponential distribution in the case of a continuous-time process). The inset in Fig. 9c shows the IEI distribution from the example process plotted with a logarithmic rate axis. Due to right censoring caused by the finite observation window, this distribution deviates at longer IEIs from the straight line that would be expected for a geometric distribution. Figure 9d shows the corresponding IDI distributions. The numerical results and the simulation results are in close agreement. Although a closed-form expression is available for the continuous counterpart to this distribution (Young and Barta 1986; Heil et al. 2007; Neubauer et al. 2009), it has been derived assuming an infinitely long observation window which avoids the effects of right censoring. This closed-form solution would therefore account poorly for any experimentally obtained interval distributions that are strongly affected by right censoring. As the observation window becomes shorter and right censoring becomes more pronounced, the mean and standard deviation of the observed intervals decrease relative to those expected for the underlying process (Nawrot et al. 2008). Because a distribution computed with the numerical method presented here includes the effects of right censoring, it can be preferable to a distribution computed from a known closed-form expression.

Although a homogeneous event process (Fig. 9a) is in equilibrium, the detection process (Fig. 9b) that arises after applying random dead times is not. The nonequilibrium nature of the detection process manifests as a detection rate that initially equals the event rate but then decreases and settles into a lower steady-state rate. The higher detection rate at the start of the observation window results from the assumption that the detector is not initially in a dead state, such that the distribution of forward recurrence times to the first detection is identical to the distribution of forward recurrence times to the first event. The lower detection rate in the steady state results from the fact that, for all detections after the first one, dead-time effects cause the probability mass in the distribution of forward recurrence times to the next detection to be shifted toward later times than in the corresponding distribution of forward recurrence times to the next event. For a homogeneous event process, the steady-state detection rate equals the inverse of the sum of the mean IEI and the mean dead time. This yields a steady-state rate of 526.3 detections/s for the example process (Fig. 9b), which has a mean IEI of (1−*p*_event_)·(Δ*t*/*p*_event_) = 0.9 ms and a mean dead time of *d*_fixed_ + *μ*_rand_ = 1 ms.

## Discussion

The numerical method presented here yields the expected distribution of intervals between detections of events in an inhomogeneous Poisson point process, assuming the detector is nonparalyzable and each detection is followed by a dead time drawn from a fixed probability distribution. There are, however, several limitations and considerations to keep in mind, as described below.

### The method models the process in discrete time

Although point processes in nature operate in continuous time, numerical computation methods necessarily treat them as though that they operate in discrete time. For a stochastic process that operates in continuous time, the IDI distribution obtained with the discrete numerical method is therefore an estimate which converges to the exact solution as the time step approaches zero. There are, however, practical limits to how brief the time step can be in the numerical method. Shortening the time step or prolonging the rate function increases the number of time points in the observation window, and the computation time of the method grows super-linearly with the number of time points. The main computational bottleneck is Eq. 8, which has a computation time that grows approximately in proportion to the cube of the number of time points. Nonetheless, to obtain an adequate description of a continuous point process, the time step in the numerical method must be sufficiently brief. At a minimum, it should be brief enough that each time step would be expected to have either zero events or one event, but not several events. It should also be brief enough that fluctuations in the rate function are not undersampled. For example, to model a process in which the rate fluctuates sinusoidally with a frequency of *f* Hz, the author suggests a time step of Δ*t* ≤ (200⋅*f*)^−1^ s, corresponding to a sampling rate of at least 200 times *f*.

### The method describes a nonequilibrium detection process

It has been shown that the numerical method can be used to compute IDI distributions for either homogeneous (Fig. 9) or inhomogeneous (Figs. 1, 2, 3, 4, 5, 6, 7, 8) event processes. In either case, the detection process is not in equilibrium, because there are transient changes in the detection rate at the start of the process before it settles into a steady state (Deger et al. 2010). This effect is most apparent for the process with a constant event rate (Fig. 9a, b), although it also occurs for the process with a periodic event rate (Figs. 4c and 2). For a process to be in equilibrium at the start of the observation window, it must have both begun and settled into its steady state prior to the start of the observation window. However, if a process began prior to the start of the observation window, then it might be in a dead state at the start of the observation window, which violates an assumption of the numerical method presented here. Adapting the method to describe an equilibrium detection process would therefore require considering the probability that each time point in the observation window is in a dead state resulting from a detection occurring prior to the observation window. The numerical method presented here is therefore suitable only for processes that begin at the start of the observation window or processes that have a negligible event probability prior to the start of the observation window. An example of such a process would be a train of spikes recorded from a neuron in response to a stimulus, provided the neuron fires no (or negligibly few) spikes in the absence of a stimulus prior to the observation window.

### The method requires that the detection probability be independent of the process history prior to the previous detection

The numerical method presented here works only for processes in which the instantaneous probability of a detection depends only on the current time point in the observation window and on the time elapsed since the most recent detection (necessary for knowing the probability that the process is still in a dead state). The detection process can be either an “ordinary renewal process” (which results if the underlying event process is homogeneous such that the intervals between detections are independent and identically distributed) or an “inhomogeneous renewal process” (which results if the underlying event process is inhomogeneous such that the distribution of intervals to the next detection will depend on the rate function following the most recent detection). The numerical method can be applied in either case, so long as the probability of detection is not influenced by the process history prior to the most recent detection. The method does not work for nonrenewal processes.

### The method operates in one direction only

Given a particular dead-time distribution, the numerical method can be used to work forward from the rate function to the IDI distribution. It is not possible, however, to work backward from the IDI distribution to the rate function that gave rise to it because the interval distribution contains little information about the timing of detections within the observation window (Turcott et al. 1994; Bi et al. 1988). Indeed, many different rate functions can give rise to virtually identical interval distributions. This can be demonstrated for a simple case by comparing a homogeneous Poisson point process to a “doubly stochastic” point process in which the event rate varies stochastically over time (Cox 1955). For sufficiently long observation windows, both processes can yield interval distributions that are essentially identical, despite the processes having different rate functions (Teich et al. 1990; Lowen and Teich 1991).

### Previous approaches

Several investigators have presented results that relate, to varying degrees, to the present work. To the author’s knowledge, however, none has presented a method to compute the IDI distribution expected for any arbitrary inhomogeneous Poisson point process of events modified by dead times drawn from any arbitrary distribution. For example, closed-form expressions have been presented to describe the IDI distribution for the dead-time-modified homogeneous (i.e., constant-rate) Poisson point process, which in the absence of right censoring is given by the convolution of the IEI distribution and the dead-time distribution (Young and Barta 1986; Li and Young 1993; Picinbono 2009; Prijs et al. 1993; Heil et al. 2007; Neubauer et al. 2009). Although Turcott et al. (1994) provide an expression for the IDI distribution of an inhomogeneous Poisson point process, it is for the very specific case in which the rate function decays exponentially and dead times are nonrandom and inversely proportional to the instantaneous detection rate. Deger et al. (2010) present a method to compute the time-dependent hazard function (their Eq. 22) for an inhomogeneous Poisson point process modified by random dead times. If converted to the corresponding probability distribution, this hazard function is equivalent to the distribution of forward recurrence times to the next detection obtained in the present study (Eq. 8). However, Deger et al. (2010) did not proceed to compute the expected IDI distribution from this result. Yakovlev et al. (2005) present a method to compute the expected IEI distribution for any arbitrary inhomogeneous Poisson point process, but not for one that has been modified by dead times. Deriving the expected IDI distribution is therefore a novel contribution of the present study.

In the neuroscience literature, there exist two common implementations for applying dead-time effects to a point process of events. One implementation treats the dead time as a random variable, as in the present study, and any events which occur during a dead time fail to be detected (e.g., Teich et al. 1978; Young and Barta 1986; Li and Young 1993; Franklin and Bair 1995; Liu et al. 2001; Heil et al. 2007; Neubauer et al. 2009; Deger et al. 2010; Peterson et al. 2014; Peterson and Heil 2018). In the alternative implementation, the dead time has the effect of transiently and deterministically reducing the rate of the process following each detection (e.g., Gray 1967; Lütkenhöner et al. 1980; Teich and Diament 1980; Gaumond et al. 1982; Johnson and Swami 1983; Jones et al. 1985; Miller 1985; Bi et al. 1988; Bi 1989; Miller and Mark 1992; Carney 1993; Prijs et al. 1993; Delgutte 1996; Johnson 1996; Berry and Meister 1998; Zhang et al. 2001; Sumner et al. 2002,2003; Meddis 2006; Zilany and Bruce 2006; Zilany et al. 2009). In this implementation, the detection rate is conceived of as the product of two independent components, one of which is the stimulus-dependent excitation function, *s*(*t*), describing the rate of the event process without dead-time effects, and the other of which is the recovery function, *r*(*t-t’*), describing how the rate is to be scaled down following a detection at time *t’* (or, equivalently, describing the probability that an event which has occurred based on the original event-rate function will be detected). In the notation of the present study, the detection rate following a detection at time *t*_*i*_ would be given by *R*_detection_(*t*_*i*+*j*_) = *R*_event_(*t*_*i*+*j*_)·*G*_dead_(*d*_*j*_), where *G*_dead_ is, for example, given by Eq. 1 but is interpreted as a recovery function rather than as a cumulative dead-time distribution. Although the difference between the two implementations can be quite small when the refractory periods are short and the rate is low, the implementations are not equivalent (Peterson et al. 2014). The results obtained using the numerical method presented here are valid only for the implementation in which the dead time is treated as a random variable. Jones et al. (1985) present a procedure to obtain the IDI distribution for the alternative implementation.

### Applications and future work

Although the numerical method is presented as though the rate function of the detection process is known from experimental observation and can therefore be used to compute the expected IDI distribution, this is not the primary application of the method envisioned by the author. After all, an investigator who has observed a process experimentally to obtain the rate function would be well positioned to simply compute an empirical IDI distribution directly from the data. Rather, the more interesting application of the method is in modeling. One can use the method to answer the question of how a given dead-time distribution will affect the IDI distribution of a process characterized by a given rate function. This is true whether the rate function assumed by the investigator describes the event process or the detection process, because the method enables each one to be obtained from the other. It is not necessary that the rate function or the dead-time distribution be representable by a closed-form expression, and it is certainly not necessary that a closed-form expression exists for either the IEI or IDI distributions. The method could also be useful in cases for which the dead-time distribution giving rise to a particular experimentally observed IDI distribution is unknown; in such a case, it can be used to model how several candidate dead-time distributions would affect the shape of the IDI distribution and thereby aid in selecting a dead-time distribution that can account well for the observed data.

Future work could include adapting the method to also account for equilibrium processes, although this would require knowledge of the rate function prior to the start of the observation window so that the probability that the process is in a dead state due to a detection occurring prior to the window can be taken into consideration. This would require that a steady state can be derived for the rate function prior to the observation window (e.g., if the function is constant or periodic). Otherwise, the equilibrium process can only be approximated using the current method by extending the observation window backward in time to allow the process to reach a steady state prior to the start of the original observation window, and then computing the IDI distribution using only the time points within the original observation window. The method can perhaps also be adapted to describe non-Poisson point processes modified by random dead times, or even multiple non-Poisson point processes which are superposed and then modified by random dead times as has been suggested previously to account for nonrenewal properties of spontaneous spike trains of auditory-nerve fibers (Peterson and Heil 2018). However, the longer-term history effects present in a nonrenewal event process would make such an extension of the method substantially more complex than the comparatively simple renewal event process presented here.

## Data Availability

Not applicable.
